# Open defecation practice and its determinants among households in sub-Saharan Africa: pooled prevalence and multilevel analysis of 33 sub-Saharan Africa countries demographic and health survey

**DOI:** 10.1186/s41182-022-00416-5

**Published:** 2022-03-29

**Authors:** Daniel Gashaneh Belay, Melaku Hunie Asratie, Fantu Mamo Aragaw, Nuhamin Tesfa Tsega, Mastewal Endalew, Moges Gashaw

**Affiliations:** 1 Department of Epidemiology and Biostatistics, Institute of Public Health, College of Medicine and Health Sciences, University of Gondar, Gondar, Ethiopia; 2 Department of Human Anatomy, College of Medicine and Health Sciences, University of Gondar, Gondar, Ethiopia; 3 Department of Women’s and Family Health, School of Midwifery, College of Medicine and Health Sciences, University of Gondar, Gondar, Ethiopia; 4 Department of Physiotherapy, College of Medicine and Health Sciences, University of Gondar, Gondar, Ethiopia; 5 Department of Environmental and Occupational Health and Safety, Institute of Public Health, College of Medicine and Health Sciences, University of Gondar, Gondar, Ethiopia

**Keywords:** Open defecation, Inequalities, Sub-Saharan Africa

## Abstract

**Background:**

Open defecation facilitates the transmission of pathogens that cause diarrheal diseases, which is the second leading contributor to the global burden of disease. It also exposed hundreds of millions of girls and women around the world to increased sexual exploitation. Open defecation is more practice in sub-Saharan African (SSA) countries and is considered an indicator of low socioeconomic status. However, there is little evidence on the pooled prevalence and factors contributing to open defecation practice among households in SSA.

**Objectives:**

This study aimed to assess the pooled prevalence, wealth-related inequalities, and other determinants of open defecation practice among households in sub-Saharan Africa.

**Methods:**

Demographic and Health Survey data sets of 33 SSA countries with a total sample of 452,281 households were used for this study. Data were weighted, cleaned, and analyzed using STATA 14 software. Meta analyses were used to determine the pooled prevalence of open defecation practice among households in SSA. Multilevel analysis was employed to identify factors contributing to open defecation practice among households in SSA. Moreover, concentration index and graph were used to assess wealth-related inequalities of open defecation practice. The associations between dependent and independent variables were presented using adjusted odds ratios and 95% confidence intervals with a *p* value of < 0.05.

**Results:**

The pooled prevalence of open defecation practice among households in sub-Saharan African countries was 22.55% (95%CI: 17.49%, 27.61%) with *I*^2^ = 99.9% and ranges from 0.81% in Comoros to 72.75% in Niger. Individual level factors, such as age, educational attainment, media exposure, wealth status, and access to drinking water, as well community level factors, such as residence, country income status, and region in SSA, had a significant association with open defecation practice. The concentration index value [C = − 0.55; 95% CI: − 0.54, − 0.56] showed that open defecation practice was significantly disproportionately concentrated on the poor households (pro-poor distribution).

**Conclusions:**

Open defecation practice remains a public health problem in sub-Saharan Africa. Individual level factors, such as age, educational attainment, media exposure, household wealth status, and access to drinking water had an association with open defecation practice. Moreover, community level factors such as residence, country income status and region in SSA have a significant effect on open defecation. There is a significantly disproportional pro-poor distribution of open defecation practice in SSA. Each country should prioritize eliminating open defecation practices that focused poorest communities, rural societies, and limited water access areas. Media exposure and education should be strengthened. Moreover, public health interventions should target to narrow the poor-rich gap in the open defecation practice among households including provisions of subsidies to the poor. Policymakers and program planners better use this evidence as preliminary evidence to plan and decide accordingly.

## Background

The disposal of human faces in the fields, bushes, forests, open bodies of water, beaches, and other open spaces is called open defecation [1]. According to World Health Organization (WHO) and United Nations Children’s Fund (UNICEF) Joint monitoring program (JMP) 2021 reports, 494 million people practice open defecation [2]. Most of (92%) these people lived in rural areas and nearly half of them lived in sub-Saharan Africa [2]. There was a nearly 50% decrement (from 23 to 12%) in open defecation practice in Central and Southern Asia from 20,015 to 2020, whereas in sub-Saharan African countries, it decreased only from 22 to 18% [3].

Diarrheal disease is the second major cause of death in children under the age of five, causing 1.7 million morbidities and 760,000 deaths every year globally [4]. In Africa, it is also one of the main causes of death in under-five children [5]. Poor sanitation is a serious public health issue that has been related to several undesirable health outcomes, including diarrheal diseases and trachoma [6]. The practice of open defecation (OD) aids in the transmission of microorganisms that cause diarrheal diseases [7], with children being the most vulnerable [8]. A study showed that the prevalence of diarrhea was four times higher among OD practice communities as compared to OD-free areas [9]. Open defecation also the risks of exposing hundreds of millions of girls and women around the world to increased sexual exploitation and lack of privacy when they are menstruating [10].

Studies showed that the majority of OD practices were taken place in rural areas of low-income countries [11]. Other factors such as financial status of the household [12, 13], household size [13], occupation [13], and region [14] had an association with open defecation.

Interventions to improve human excreta disposal facilities have been demonstrated to be successful in preventing diarrheal diseases at their most important source by preventing human fecal contamination of water and soil [5, 15]. According to the 2030 Sustainable Development Agenda, no child should die or get sick as a result of drinking contaminated drinking water, and/or being exposed to other people's excreta [16].

However, with these interventions, the practice of OD in sub-Saharan Africa (SSA) is not significantly decreased [2, 17, 18] as a result, children's death due to diarrheal disease is common [19]. However, there is little evidence on the pooled prevalence and factors contributing to OD practice among households in SSA. Therefore, this study amid to assess the pooled prevalence, wealth-related inequalities, and other determinants of OD practice among households in SSA. Understanding these different patterns of inequality is an important first step in devising appropriate strategies to reduce them [20]. It is also critical to understand what factors influence the pace of improving sanitation and reducing diarrhea morbidity and mortality caused by the lack of sanitation.

## Methods

### Study setting, and period

This study was conducted among 33 SSA countries. The sub-Saharan is the area in the continent of Africa that lies south of the Sahara and consists of four vast and distinct regions, i.e., Eastern Africa, Central Africa, Western Africa, and Southern Africa. Together, they constitute an area of 9.4 million square miles and a total population of 1.3 billion inhabitants [21]. Recent standard DHS data set of SSA countries within 10 years (2010–2020) were our data source. To get a representative sample of recent standard DHS data from each region of SSA, 10 years of DHS data (starting from 2010) were taken. The surveys are nationally representative of each country and population-based with large sample sizes [22].

A total of thirty-three SSA countries were represented for this study in the four regions. In Eastern Africa, eleven countries (Burundi, Comoros, Ethiopia, Kenya, Malawi, Mozambique, Rwanda, Tanzania, Uganda, Zambia, Zimbabwe), in Southern Africa three countries (Lesotho, Namibia, South Africa), in Central Africa six countries (Angola, Cameroon, Chad, the Democratic Republic of the Congo, Republic of the Congo, Gabon), and in Western Africa thirteen countries (Benin, Burkina Faso, Ivory Coast, Gambia, Ghana, Guinea, Liberia, Mali, Niger, Nigeria, Senegal, Sierra Leone, Togo) were included for this study.

### Population

Of the total of 47 countries located in SSA, only 41 countries had Demographic and Health Survey reports. From these, five countries, namely, Central Africa Republic (DHS report 1994/95), Eswatini (DHS report 2006/07), Sao Tome Principe (DHS report 2008/09), Madagascar (DHS report 2008/09), and Sudan (DHS report 1989–90) have a survey report before the 2010 survey year and excluded from further analysis. Moreover, three countries (Botswana, Mauritania, and Eritrea) were excluded due to the DHS data set not being publicly available. Finally, a total of 33 sub-Saharan African countries were included in this study.

All households which were found across 33 SSA countries during the survey period were our source population. Whereas households assessed for sanitation facilities during each survey across 33 SSA countries were our study population. Finally, the analysis contained a total weighted sample of 452,281 households.

### Sampling method

The most recent standard census frame was used in all of the surveys conducted in the selected countries. Typically, DHS samples are stratified by administrative geographic region and by urban/rural areas within each region.

DHS sample designs are usually two-stage probability samples drawn from an existing sample frame. Stratification was achieved by separating every geographical region in the countries into urban and rural areas. In the first stage of sampling, Enumeration Areas (EAs) were selected with probability proportional to size within each stratum. In selected EAs, following the listing of the households, a fixed number of households is selected by equal probability systematic sampling The detailed sampling procedure was available in each DHS reports from the Measure DHS [22].

The household records (HR files) data sets were used. Weighted values were used before using the DHS data set to restore the representativeness of the sample data. Since the overall probability of selection of each household is not constant. DHS guideline set four sampling weighting methods and from that, we used the household sampling weight (hv005). Sample weights were generated by dividing (hv005) by 1,000,000 before use to approximate the number of cases [23]. However, there was no change in the value of the total sample size after weighting which was 452,281 (Table 1).

**Table 1:** Sample size determination in the study of pooled prevalence of open defecation and determinants among households in sub-Saharan Africa 2010–2020 DHS

Sub-Saharan Africa Countries with Recent DHS report from 2010/11 to 2019/20				
Regions	Countries	Standard DHS year	Sample size (*n*)	Percentage (%)
East Africa countries	Burundi	2016/17	15,977	3.53
	Comoros	2012	4482	0.99
	Ethiopia	2016	16,650	3.68
	Kenya	2014	36,430	8.05
	Malawi	2015/16	26,361	5.83
	Mozambique	2015	7169	1.59
	Rwanda	2019/2020	12,949	2.86
	Tanzania	2015/16	12,563	2.78
	Uganda	2016	19,588	4.33
	Zambia	2018	12,831	2.84
	Zimbabwe	2015	10,534	2.33
	Subtotal		175,534	38.81
	Angola	2015/16	16,109	3.56
	Cameroon	2018	11,710	2.59
Central Africa countries	Chad	2014/15	17,233	3.81
	DR Congo	2013/14	18,171	4.02
	Congo	2011/12	11,632	2.57
	Gabon	2012	9755	2.16
	Subtotal		84,610	18.71
	Benin	2017/18	14,156	3.13
	Burkina Faso	2011	14,424	3.19
	Ivory Coast	2011/12	9686	2.14
	Gambia	2019/20	6549	1.45
	Ghana	2014	11,835	2.62
West Africa countries	Guinea	2018	7912	1.75
	Liberia	2019/20	9068	2
	Mali	2018	9510	2.1
	Niger	2012	10,750	2.38
	Nigeria	2018	40,427	8.94
	Senegal	2019	4538	1
	Sierra Leone	2019	13,399	2.96
	Togo	2013/14	9549	2.11
	Subtotal		161,803	35.77
Southern Africa countries	Lesotho	2014	9402	2.08
	Namibia	2013	9849	2.18
	South Africa	2016	11,083	2.45
	Subtotal		30,334	6.71
Total sample size			452,281	100%

### Study variables

The outcome variables of the study were open defecation which includes households with a lack of sanitation facility, defecating on bush or field [24]. The independent variables considered for this study were categorized as individual-level variables, such as age, sex, marital status, and educational attainment of household head, household family size, media exposure status of the households, and household wealth index. Whereas community level variables, such as place of residence, region in sub-Saharan Africa, survey year, and country income level (Table 2).

**Table 2:** List of the independent variables used in the study with their measurement descriptions

Level	Variables	Measurements
Individual level variables	Age	The age of women categorized as 11–25, 26–40, 41–60, and > 60
	Sex	Sex of the household head categorized as male and female
	Education level	Educational attainment is categorized as uneducated, primary, secondary, and above
	Marital status	The marital status of the household is categorized as married and not married
	Family size	Categorized as 1–3, 4–6, and 7 and above
	Media exposure	A composite variable obtained by combining whether a respondent listens to the radio, and watch television with a value of “0” if women were not exposed to at least one of the two media, and “1” if a woman has access/exposure to at least one of the two media [25]
	Wealth index	The data sets contained a wealth index that was created using principal components analysis coded as poorest, poorer, middle, richer, and richest in the DHS data set. For this study, we recorded it in three categories poor (including poorer and poorest), middle and rich (includes richer and richest)
	Access to a drinking water source	Basic drinking services: drinking water from an improved source, provided collection time is not more than 30 min for a round trip, including queuing [18]. On the other side limited drinking services: drinking water from an improved source for which collection time exceeds 30 min for a round trip, including queuing [18]
Community-level variables	Residency	Urban or rural based on where the household lives in the data set was used without change
	Region	The regions in sub-Saharan Africa were categorized as Eastern Africa, Central Africa, Western Africa, and Southern Africa
	Countries income level	The countries income status was categorized as low income, lower middle income, and upper-middle-income country based on the World Bank List of Economies classification since 2019 [26]. World Bank calculated country income based on Gross National Income (GNI) per capita, which categorized as low income $1,025 or less; lower middle income, $1,026–3,995, upper middle income $3,996–12,375,and high income $12,375 or more [26]
	DHS survey year	Survey year means the recent standard DHS data collection period of each country from 2010 to 2020. Categorized as the survey years 2010–2014 and 2015–2020

### Data management and analysis

This study was performed based on the DHSs data obtained from the official DHS measure after permission was obtained. The set of household data (HR) data was used to extract the outcome and the independent variables. The data clearance, descriptive, and summary statistics were conducted using STATA version 14 software. Before we conduct any statistical analysis, the data were weighted for the sampling probabilities using the weighting factor to restore the representativeness of the survey and to get reliable statistical estimates.

The pooled estimate of open defecation practice among households in SSA and sub-regions was estimated using a *metan* STATA command. It was determined using the proportion of OD of each SSA country and the standard error which was calculated from the proportion and sample size in each country. Then further subgroup analyses were done to minimize the heterogeneity between studies using region in SSA, level of income of the country, and the DHS survey year.

### Mixed effect analyses and model building

Since the DHS data have a hierarchical structure, where households are nested within a cluster/EAs, which violates the assumption of independence of observations and equal variance across clusters, mixed effect models which include both fixed and random effects were used to assess the clustering effect of open defecation usage among 33 sub-Saharan African countries.

The fixed effects were used to estimate the association between the likelihood of OD and explanatory variables at both individual and community levels. In the multivariable analysis, the associations between dependent and independent variables were presented using adjusted odds ratios and 95% confidence intervals with a *p* value of < 0.05.

Random-effects were used to estimate a measure of variation and estimated using the Interclass Correlation Coefficient (ICC), Median Odds Ratio (MOR), and Proportional Change in Variance (PCV).

The ICC reveals the variation of OD between clusters is calculated as; $\mathrm{ICC}=\frac{\mathrm{VC}}{\mathrm{VC}+3.29}*100\%$, where VC = cluster level variance.

The MOR is defined as the median value of the odds ratio between the area at the lowest risk and at the highest risk when randomly picking out two clusters.

MOR = exp.[√(2 × VC) × 0.6745], or ${{ \mathrm{MOR}=e}^{0.95}}^{\sqrt{\mathrm{VC}}}$ where VC is the cluster level variance.

The PCV shows the variation in OD among households explained by both individual and community level factors. $\mathrm{PCV}=\frac{V\mathrm{null}-\mathrm{VC}}{V \mathrm{null}}*100\%$ where Vnull = variance of the initial model, and VC = cluster level variance of the next model [27–29].

In general, in mixed-effect analysis, four models were fitted. The first was the null model containing only the outcome variables which were used to check the variability of OD in the cluster. The second and the third multilevel models contain household-level variables and community-level variables, respectively, whereas in the fourth model both household and community level variables simultaneously were fitted with the OD. Model comparison was done using the likelihood ratio and deviance test and the model with the highest likelihood and the lowest deviance was selected as the best-fitted model [27–29].

### Concentration index and graph analyses

The concentration index and graph approach are used to examine socioeconomic inequalities in health outcomes [30, 31]. The concentration curve is used to identify whether socioeconomic inequality in some health variables exists and whether it is more pronounced at one point. It displays the share of health outcomes accounted for cumulative proportions of individuals in the population ranked by wealth status from the poorest to the richest [31, 32]. This study's health outcome variable was the cumulative proportion of open defecation practice, whereas the wealth status of the households was ranked the poorest to the richest (poorest, poorer, middle, richer, and richest).

The concentration curve would be a 45^0^ line indicating the absence of inequity. Whereas, the concentration curve lying above and below the equality line (45^0^) indicated that OD practice is disproportionately concentrated between poor and rich, respectively [33]. The greater the degree of inequity, the more the concentration curve diverged from the diagonal line [31]. Twice the area between the concentration curve and the diagonal line is the concentration index [32, 34]. It ranges from − 1 to + 1 and the sign indicates the direction of the relationship between the health variable (OD practice) and the distribution of living standards (wealth status) [31, 35].

## Result

### Sociodemographic characteristics of the study population

A total weighted 452,281 households in 33 SSA countries were included in this study. From these, nearly three fourth 328,270 (72.58%) of the household heads were males. Nearly three-fifths (61.48%) of the study participants were living in rural areas and of them about one-third (31.87%) practice OD. Nearly one-third (32.85%) of the head of household had no formal education and from them, two-fifths (38.17%) practiced OD. From the total 150,716 (35.19%) households, 34.54% were practice OD (Table 3).

**Table 3:** Socio-demographic characteristics of the study households with open defecation and determinants among households in sub-Saharan Africa 2010–2020 DHS

Variables	Categories	Open defecation		Total weighted frequency (%) *n* = 452,281
		Yes (%) *n* = 101,318 (22.55)	No (%) *n* = 350,963 (77.45)	
*Individual level factors*				
Age of household head (years)	11–25	10,067 (24.53)	30,970 (75.47)	41,037 (9.07)
	26–40	37,403 (21.38)	137,556 (78.62)	174,959 (38.69)
	41–60	26,799 (21.44)	99,370 (78.76)	126,169 (27.9)
	> 60	27,038 (24.56)	83,042 (75.44)	110,081 (24.34)
Sex of household head	Male	74,456 (22.68)	253,814 (77.32)	328,270 (72.58)
	Female	26,862 (21.66)	97,149 (78.34)	124,011 (27.42)
Educational attainment of household head	No education	56,653 (38.17)	91,768 (61.83)	148,421 (32.85)
	Primary education	27,735 (19.47)	114,720 (80.53)	142,455 (31.53)
	Secondary and above	16,794 (10.43)	144,193 (89.57)	160,987 (35.63)
Marital status of head of household	Married	72,415 (24.49)	223,269 (75.51)	295,684 (65.38)
	Not married	28,904 (18.46)	127,693 (81.54)	156,597 (34.62)
House hold family size	1–3	33,797 (20.74)	129,132 (79.26)	162,929 (36.02)
	4–6	40,857 (22.47)	140,972 (77.53)	181,830 (40.20)
	7 and above	26,663 (24.8)	80,858 (75.2)	107,522 (23.77)
Media exposure	No	58,231 (32.95)	118,481 (67.05)	176,712 (39.08)
	Yes	43,062 (15.63)	232,403 (84.37)	275,465 (60.92)
Wealth index	Poor	71,472 (40.30)	105,900 (59.70)	177,372 (39.22)
	Middle	19,007 (21.32)	70,131 (78.68)	89,138 (19.71)
	Rich	10,839 (5.83)	174,932 (94.17)	185,771 (41.07)
Access to drinking water	Basic	45,197 (16.42)	230,026 (83.58)	275,224 (60.86)
	Limited	56,080 (31.69)	120,912 (68.31)	176,993 (39.14)
*Community-level variables*				
Residence	Urban	12,697 (7.29)	161,541 (92.71)	174,239 (38.52)
	Rural	88,621 (31.87)	189,421 (68.13)	278,042 (61.48)
Region in SSA	Central Africa	21,557 (25.48)	63,052 (74.52)	84,610 (19.76)
	East Africa	19,316 (11.88)	143,269 (88.12)	162,585(37.97)
	West Africa	52,059 (34.54)	98,657 (65.46)	150,716 (35.19)
	Southern Africa	7371 (24.30)	22,963 (75.70)	30,334 (7.08)
Country income level	Lower income	66,242 (27.89)	171,254 (72.11)	237,496 (55.46)
	Lower middle	24,184 (16.8)	119,769 (83.2)	143,953 (33.61)
	Upper middle	9878 (21.11)	36,917 (78.89)	46,796 (10.93)
Survey year	2010–2014	53,271 (32.59)	110,172 (67.41)	163,443 (36.14)
	2015–2020	48,047 (16.63)	240,791 (83.37)	288,838 (63.86)

### The pooled prevalence of open defecation among households in sub-Saharan Africa

**Fig. 1:**
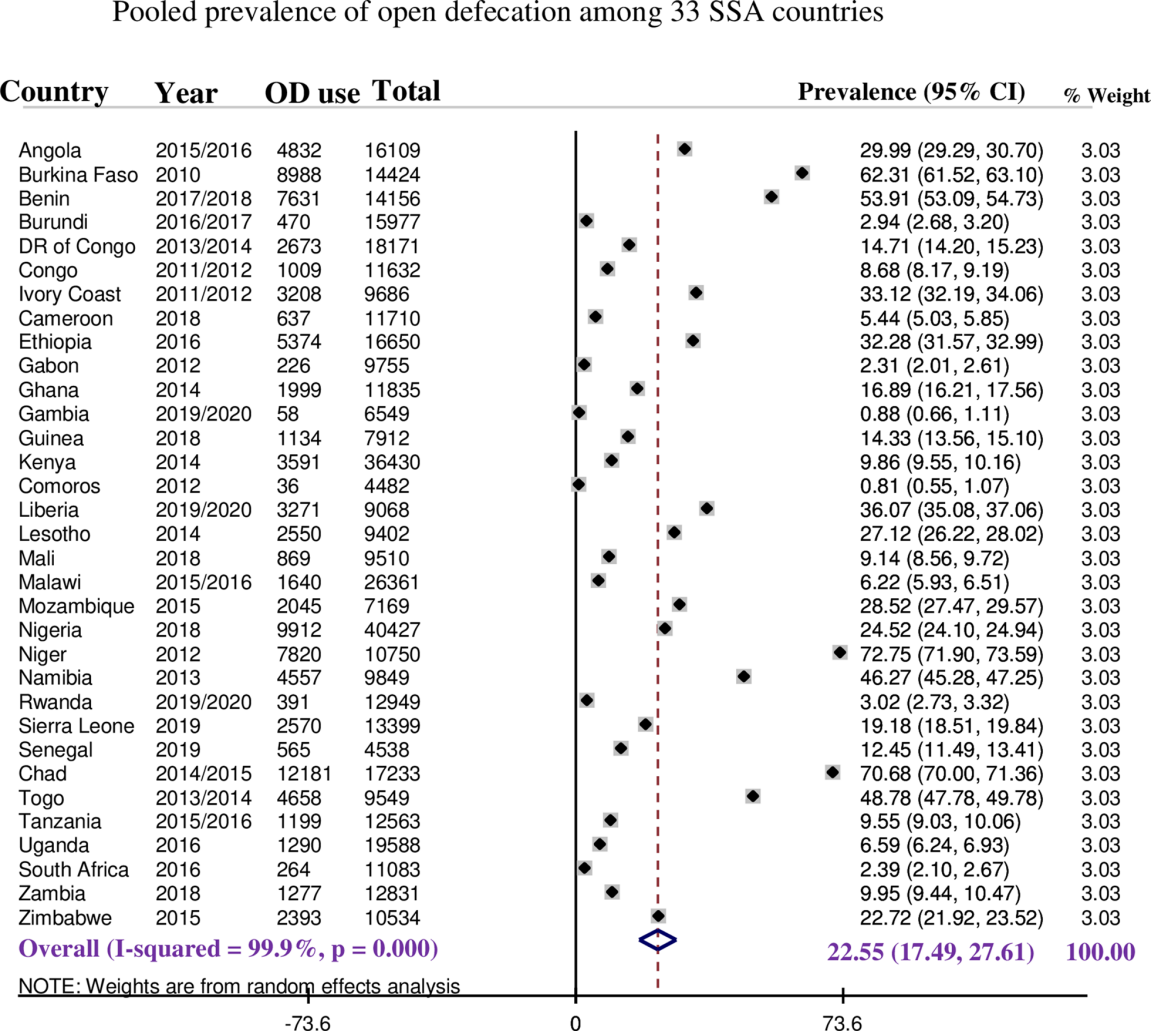
Forest plot showed that, the pooled prevalence of open defecation among households in SSA

The overall pooled estimate of open defecation among households in sub-Saharan African countries was 22.55% (95%CI: 17.49%, 27.61%) with *I*^2^ = 99.9% and ranges from 0.81% (95%CI: 0.55, 1.07) in Comoros to 72.75% (95%CI: 71.90, 73.59) in Niger (Fig. 1).

Since the *I*^2^ value was large, which shows the true variabilities (the variability not by chance) of OD among households in 33 SSA countries, then to treat this heterogeneity effect further subgroup analyses were performed based on the region in SSA, level of income of the country and the DHS survey year. Based on subgroup analysis using regions in SSA, the pooled prevalence of OD ranges from 12.02% (95%CI: 8.12%, 15.93%) in Eastern Africa across 11 countries to 31.10% (95%CI: 18.71%, 43.49%) among 13 West African countries. Moreover, the pooled prevalence of OD across countries' income levels was determined. Among 21 low-income level countries, the pooled prevalence of OD was 25.13% (95%CI: 17.73%, 32.53%), whereas it was 20.23% (95%CI: 6.50%, 33.97%) across 4 upper middle-income countries**.** In addition, the pooled prevalence in 15 countries whose DHS survey was conducted before and in 2015 was 31.03% (95%CI: 20.11%, 41.95%), whereas it was 15.48% (95%CI: 10.85%, 20.12%) in 18 countries whose DHS survey after 2015 (Table 4).

**Table 4:** Subgroup analyses of open defecation among households in SSA

Subgroup	Categories	Number of countries	Prevalence	*X*^2^-heterogeneity	*I*-squared	*p* value
Region in SSA	Central Africa	6	21.92%	36,403.16	99.9%	< 0.001
	Western	13	31.10%	65,117.78	100.00%	< 0.001
	Eastern	11	12.02%	12,148.42	99.9%	< 0.001
	Southern	3	25.55%	9029.26	99.9%	< 0.001
Income status of the countries	Lower income	21	25.13%	1.1*10^5^	100.00%	< 0.001
	Lower middle income	8	16.94%	7851.62	99.9%	< 0.001
	Upper middle income	4	20.23%	12,046.93	99.9%	< 0.001
DHS released year	Released before and in 2015	15	31.03%	88,842.91	99.9%	< 0.001
	Released after 2015	18	15.48%	38,883.39	100%	< 0.001
Total		33	22.55%	1.4*10^5^	99.9%	< 0.001

### Multi-level analysis of factors associated with open defecation among households in sub-Saharan Africa

In random effect analysis, the ICC in the null model showed that about 36% of the variations of OD practices among study households were attributed to the difference at the cluster level. The MOR value in the null model also revealed that the median odds of using OD between the highest open defecate clusters and the lowest open defecate clusters was 3.64.

Furthermore, the PCV valve in the final model (0.054) indicates the variation in the OD usage among study households was explained by both the individual and community level factors simultaneously. Model comparison/fitness was done using loglikelihood and deviance test, then the last model (Model III) has the highest log-likelihood and the lowest deviance and was taken as the best-fitted model (Table 5).

**Table 5:** Multi-level analysis of factors associated with open defecation practice among households in SSA from 2010 to 2020 DHS

Variables	Categories	Null model	Model I AOR [95% CI]	Model II AOR [95% CI]	Model III AOR [95% CI]
Age of household head (years)	11–25		1.00	–	1.00
	26–40		0.84 [0.81, 0.86]	–	0.97 [0.94, 1.00]
	41–55		0.72 [0.70, 0.74]**	–	0.73 [0.71, 0.75]*
	> 56		0.60 [0.59, 0.62]**	–	0.65 [0.63, 0.67]**
Sex of household head	Male		1.00	–	1.00
	Female		0.77 [0.75, 0.78]	–	0.99 [0.97, 101]
Educational attainment of household head	No education		1.00	–	1.00
	Primary education		0.38 [0.37, 0.39]**	–	0.67[0.66, 0.69]**
	Secondary and above		0.29 [0.29, 0.30]**	–	0.43 [0.42, 0.44]**
Media exposure	No		1.00	–	1.00
	Yes		0.86 [0.85, 0.87]**	–	0.70 [0.69, 0.71]**
Wealth index	Poor		1.00	–	1.00
	Middle		0.47 [0.45, 0.47]*	–	**0.43 [0.42, 0.44**]***
	Rich		0.13 [0.12, 0.13]***	–	**0.14 [0.13, 0.14**]***
Access to drinking water	Basic		1.00	–	1.00
	Limited		1.78[1.75, 1.81]*	–	1.46 [1.44, 1.49]*
*Community-level variables*					
Residence	Urban		–	1.00	1.00
	Rural		–	9.36 [9.15, 9.58]**	2.75 [2.68, 2.83]*
Region in SSA	Central Africa		–	1.00	1.00
	East Africa		–	0.41 [0.39, 0.42]*	0.48 [0.46, 0.49]*
	West Africa		–	2.23 [2.17, 2.29]**	2.72 [2.64, 2.80]**
	Southern Africa			0.99 [0.96, 1.02]	1.33 [1.27, 1.38]***
Country income level	Lower-income		–	1.00	1.00
	Lower middle		–	0.42 [0.41, 0.43]***	0.34 [0.33, 0.35]**
	Upper middle			**0.60 [0.54, 0.66]**	0.28 [0.22, 0.33]**
Survey year	2010–2014		–	1.00	1.00
	2015–2020		–	0.38 [0.37, 0.38]***	0.32 [0.31, 0.32]*
*Random effects*					
	VA	1.85	1.76	1.82	1.75
	ICC	0.36	0.35	0.36	0.34
	MOR	3.64	3.53	3.60	3.49
	PCV	Reff	0.048	0.016	0.054
*Model comparison*					
	Loglikelyhood ratio	− 231,079	− 188,299	− 178,129	− 157,098
	Deviance	4,621	3,765	3,562	3,141
	Mean VIF	–	1.34	1.82	1.78

Bold value variables which have significant association with open defecation practice

*ICC* inter cluster corrolation cofficent, *MOR*  median odds ratio, *PCV*  proportional change in variance, *AOR*  adjusted odds ratio, *CI*  confidence intervalm, *Com. Media*  community media usage, *Com. Poverty*  community poverty status

*P-value < 0.05, **P-value < 0.01, ***P-value < 0.001

In fixed-effect analysis, as the age of household head increase to 26–40 and ≥ 60, the odds of OD usage decrease by 27% [AOR = 0.73; 95%CI; 0.71, 0.75] and 34% [AOR = 0.65; 95%CI; 0.63, 0.67], respectively. The odds of using OD decreases by 43% and 57%, as the head of household educational status increases to primary and above primary educational status [AOR = 0.67;95%CI;0.66, 0.69] and [AOR = 0.43;95%CI; 0.42,0.44], respectively.

Households who have media exposure were 30% less likely to use OD as compared to none exposed [AOR = 0.70;95%CI; 0.69, 0.71]. Peoples who live in rural households were 2.75 times more likely to use OD as compared to urban [AOR = 2.75; 95%CI; 2.68, 2.83]. Having middle and high wealth status of the households were 0.43% and 0.14% less likely to have OD as compared to poor households [AOR = 0.43;95%CI; 0.42,0.44] and [AOR = 0.14;95%CI; 0.13, 0.14], respectively. Households who have unimproved drinking water were 1.46 times more likely to use OD than having improved drinking water [AOR = 1.46; 95%CI; 1.44,1.49].

Living in the West Africa region were nearly three times more likely to use OD, but living in Eastern Africa region were 52% less likely to practice it as compared to living in Central Africa regions, [AOR = 2.758;95%CI; 2.64, 2.80] and [AOR = 0.48;95%CI; 0.46, 0.49], respectively (Table 5).

### Wealth related inequality of open defecation

**Fig. 2:**
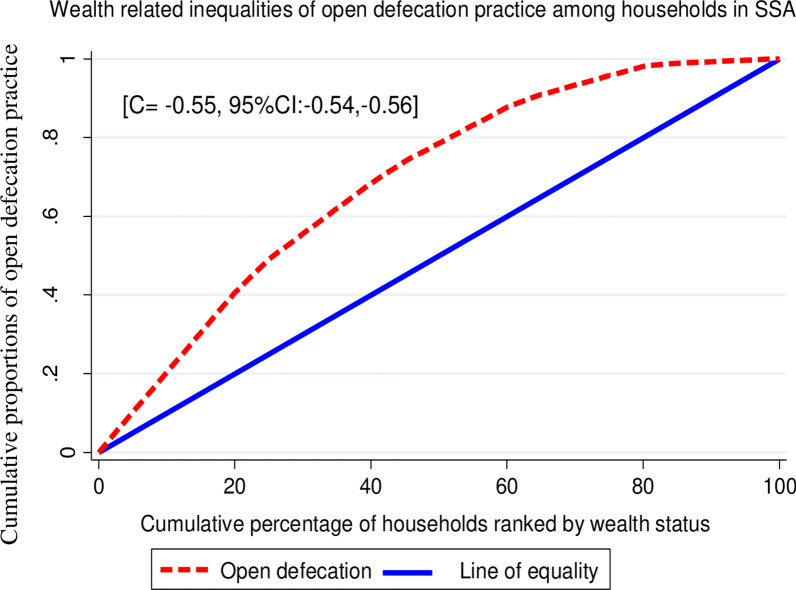
Wealth-related inequality of open defecation practice 33 sub-Saharan African countries

In this study, the wag staff normalized concentration index (*C*) and curve were done to assess the wealth-related inequality of OD practice among households in SSA. The result showed that OD was significantly disproportionately concentrated on the poor households (pro-poor distribution) with [*C* = − 0.55; 95% CI: − 0.54, − 0.56], which means that when households income status becomes lowest the burden of practicing OD is increasing. The graph in Fig. 2 also showed that the distribution line of OD is above the line of equality. This shows that OD among households in SSA was disproportionately concentrated on the poor household (pro-poor distribution) (Fig. 2).

## Discussion

This study was conducted to assess the pooled prevalence and determinants of open defecation among households in SSA. Based on this the pooled prevalence of OD practice among households of 33 SSA countries was 22.55% (95%CI: 17.49%, 27.61%). This is in line with a report by the Joint Monitoring Program (JMP) of WHO and UNICEF 2021 report in sub-Saharan Africa (18%) [2]. However, this study is lower than studies in India (39.91% and 43%) [36, 37] and, higher than a JMP 2021 report worldwide (6%), and Central and Southern Asia (12%) [2] of households practicing OD. This might be due to the difference in government commitments and involvement of different community initiative programs, which have a better approach toward the reduction of OD practice and the achievement of the desired sanitation program [12, 38]. Having household money constraints to build the sanitation facilities is also another reason to practice it [37]. On the other hand, having a toilet facility at home may not be a guarantee to use toilet facilities [13]. The community accustomed to it as the old habit are also another reason for more practicing OD [37].

In this study as the age of household head increases, the chance of OD practice becomes decreases. This is supported by studies in rural North India and Tanzania, OD practice decreases sharply among the oldest household members [39, 40]. The study in Indonesia is also showed that OD practice increasing among adults [41]. This might be due to that, as the age increases people on average are unable to move more freely outside their homes. On the other side, disability or incontinence mostly occurs in the advanced age group, which makes OD difficult or impractical [39].

In this study OD practice decrease as the educational status of the household head increase. This is supported by a study in a systematic review and meta-analysis in Ethiopia [42], a study in Tanzania [43], Nigeria [44], and Ghana [13]. This might be because educated household heads have a relatively better understanding of the relevance of having sanitation facilities and the effects of OD practice. Moreover, a higher level of education status increases the probabilities of income earning capacity of households, which is the main constrain to constructing a toilet facility [37, 45].

Households that have limited access to drinking water were more likely to use OD. This is supported by a study in Dangilla Ethiopia, which showed that having limited water access has an association with OD practice [9]. This is could be explained by the fact that households having water shortages could not keep their hygiene and might not have water for toilet usage.

In our finding, households who have media exposure were less likely to use OD. It is supported by a study in India [46], in Nigeria [47] which showed that using mass media, social media, and community-based media was important for the prevention of OD practice. Exposure to mass media increases awareness about the impacts of open defecation and enables a better internalize the benefits of using a toilet [44, 46].

In this study, having middle and high wealth status of the household, as well as households from lower-middle and upper middle-income level countries, were less likely to have OD as compared to poor households and households from lower-income level. This is in line with a study in Ethiopia [4], Nigeria [44], and Gahanna [13]. The majority of OD practices have been taking place in low-income countries [11]. However, in contrast to other studies, the prevalence of OD in upper middle-income countries was higher than in the lower middle-income countries in this study. This might be due to a small number of countries eventually a small sample sizes included in upper middle-income countries as compared to lower middle-income.

The concentration index and graph in this study also revealed that OD was significantly disproportionately concentrated in poor households. This is in line with a study in Tanzania, where a pro-poor distribution of OD practice [40]. Studies showed that there are economic inequalities of OD practices between the poorest and richest households [14]. Absolute sanitation inequalities are greatest in countries such as Pakistan with the largest spread between the richest and the poorest [20]. Countries that practiced OD most widely are those with high levels of poverty [13, 20]. A study showed that per capita aid disbursement for sanitation had a strong relationship to OD reduction in low-income countries [11].

In this study, rural households were more likely to use OD as compared to urban. This is in line with WHO reports [14], a study done in Nigeria [44], India [48], and Nepal [49]. This might be due to an unequal distribution of power and limited access to infrastructure, information, and income which leads to poor practices of OD and limited sanitation in rural residences [44].

The main strength of this study was the use of the weighted nationally representative data with a large sample which makes it representative at the national. Therefore, it can be generalized to all households during the study period in SSA countries. Moreover, the use of pooled estimation and a multilevel model took into account the nested nature of the DHS data and the variability within the countries to get a reliable estimate and standard errors. Another strength of this study was estimating the pooled estimate of OD practice in sub- Saharan Africa and sub-regions will give invaluable information for region-specific intervention. However, it is not free of limitations. The heterogeneity of the pooled estimate of OD was not managed using further subgroup analysis. Moreover, since we use the secondary data recall biases and social desirability biases might be expected.

## Conclusions

Open defecation practice remains a public health problem in sub-Saharan Africa. Individual level factors, such as being aged, having higher educational attainment, having media exposure, and having middle and higher household wealth status had a preventive effect for OD practice. However, having limited access to drinking water had a positive association with it. Moreover, community level factors, such as living in rural residences, and living in West African countries had a positive association with OD practice whereas living in East Africa and living in lower-income and lower middle income have a preventive effect for OD. There is a significantly disproportional pro-poor distribution of OD practice in SSA which means that its distribution favors the poor households. Each country should prioritize eliminating OD that focused poorest communities, rural societies, and limited water access regions. Media exposure and education should be strengthened. Moreover, public health interventions should target to narrow the poor-rich gap in the OD practice among households. Policymakers and program planners better use this evidence as preliminary evidence to plan and decide accordingly.

## Data Availability

Data is available online from the “measures DHS program” and taken after writing a concept note and getting permission to use it. Anyone can access this data set by registering through this website https://dhsprogram.com/data/new-user-registration.cfm. You can access the data set through the following my data set account https://dhsprogram.com/data/dataset_admin/login_main.cfm?CFID=10818526&CFTOKEN=c131014a480fe56-4E0C6B7F-F551-E6B2-5081744BEE982E82. The following under quotation sentences are the direct instructions, and additional information from DHS programs to use the data set. “Before you can download data sets, you must register as a DHS data user. Data set access is only granted for legitimate research purposes. Learn more about data restrictions, why we require registration, how to request access or view a list of available datasets”.

## Abbreviations

AOR: Adjusted odds ratio
CI: Confidence interval
DHS: Demographic and Health Survey
HR: Household record
JMP: Joint Monitoring Program
OD: Open defecation
SDG: Sustainable development goal
WHO: World Health Organization
UNICEF: United Nations Children’s Fund
